# Systemic Lupus Erythematosus (SLE)-Associated Jaccoud’s Arthropathy

**DOI:** 10.7759/cureus.67864

**Published:** 2024-08-26

**Authors:** Rafael Sanchez, Lovekumar Vala, Riya Dhadhal, Odalys Frontela, Jose Aldrich

**Affiliations:** 1 Department of Internal Medicine, Larkin Community Hospital Palm Springs Campus, Hialeah, USA; 2 Department of Internal Medicine, Shantabaa Medical College, Amreli, IND; 3 Department of Internal Medicine, Surat Municipal Institute of Medical Education and Research, Surat, IND; 4 Department of Rheumatology, Larkin Community Hospital Palm Springs Campus, Hialeah, USA

**Keywords:** jaccoud’s arthropathy, sle and rheumatoid arthritis, s: sle, clinical rheumatology, general internal medicine

## Abstract

Jaccoud’s arthropathy (JA) is a chronic deforming arthropathy, initially linked to rheumatic fever, now more commonly associated with systemic lupus erythematosus (SLE). We report a case of a 27-year-old male presenting with a four-month history of joint pain in the bilateral hands and feet, accompanied by stiffness but no swelling, erythema, or fever. Physical examination revealed flexion deformities, ulnar deviation at the metacarpophalangeal joints, and hyperextension at the proximal interphalangeal joints, without tenderness. Laboratory findings showed elevated anti-double stranded DNA (anti-dsDNA) antibodies and positive antinuclear antibodies (ANA), and imaging confirmed non-erosive arthropathy. Diagnosed with SLE-associated JA, the patient was treated with prednisone, diclofenac, and hydroxychloroquine, leading to significant symptom improvement and decreased anti-dsDNA antibody levels. Even though non-erosive and non-deforming arthropathy is more commonly seen in SLE, timely identification of JA as a non-erosive but deforming arthritis is crucial in differentiating SLE from rheumatoid arthritis. This case underscores the need for comprehensive evaluation and tailored therapy in complex autoimmune conditions to prevent long-term joint damage and improve patient outcomes.

## Introduction

In 1869, physician Francois-Sigismond Jaccoud identified a young patient with rheumatic fever (RF) and chronic joint deformities, later named Jaccoud's arthropathy (JA) in his honor [1]. The classical deformities associated with JA include swan neck deformity, ulnar deviation, thumb subluxation, and boutonniere deformity, similar to those seen in rheumatoid arthritis (RA) but distinct in that they are characteristically reducible with passive movement and show no joint erosions on plain radiographs. JA is most commonly associated with systemic lupus erythematosus (SLE) but has also been observed in connective tissue disorders such as systemic sclerosis (SSc), polymyositis, Sjogren's syndrome, vasculitis, and even in individuals without these conditions [1]. 

While a reducible pattern of arthropathy is common in the majority of JA cases, in more advanced stages, the joints can become fixed, sometimes referred to as severe JA, making it difficult to clinically distinguish from RA [2].

## Case presentation

This is the case of a 27-year-old male with no significant past medical history who presented to the clinic with the chief complaint of joint pain in the bilateral hands and bilateral feet. He reported having this pain for the past four months, associated with stiffness, but no associated fever, swelling, or erythema. He noted improvement in the pain after taking over-the-counter pain medications such as acetaminophen or ibuprofen.

Physical examination revealed flexion deformities and ulnar deviation at the metacarpophalangeal joints, along with hyperextension at the proximal interphalangeal joints, without associated tenderness on pressure. There was a slight reduction in the range of motion, but the patient reported no significant difficulty in daily tasks. Passive movement showed a normal range of motion in the upper hand. No significant abnormalities were noted in the rest of the physical examination. Table 1 summarizes the laboratory findings of the patient. 

**Table 1:** Laboratory Investigations Abs: antibodies; anti-dsDNA: anti-double stranded DNA; ANA: antinuclear antibodies; ANCA: anti-neutrophil cytoplasmic antibodies; C-ANCA: cytoplasmic ANCA; P-ANCA: perinuclear ANCA

Investigation	Patient’s Report	Reference Values
Hemoglobin	13.3	12.6-17.7
Antimyeloperoxidase Abs	<9.0	0.0-9.0
Anti-proteinase 3 Abs	<3.5	0.0-3.5
C-ANCA	<1:20	Neg: <1:20
P-ANCA	<1:20	Neg:<1:20
Anti-dsDNA	32	0-9
ANA Direct	Positive	
Complement C4 serum	18	9-36
Complement C3 serum	124	90-180

Imaging

X-ray of the hands showed non-erosive arthropathy bilaterally, with ulnar deviation at the metacarpophalangeal joints and hyperextension at the proximal interphalangeal joints.

Diagnosis

The diagnosis was SLE-associated Jaccoud’s arthropathy.

Treatment

The patient was initially treated with prednisone 10 mg once a day, diclofenac 100 mg as needed for pain, and hydroxychloroquine 200 mg twice a day. The prednisone dose was later tapered down to 5 mg once a day. Anti-dsDNA antibody levels started trending down from 32 (32 > 22 > 10 > 8 > 7 > 6 > 5). The patient reported improvement in joint pain and stiffness.

## Discussion

**Figure 1:**
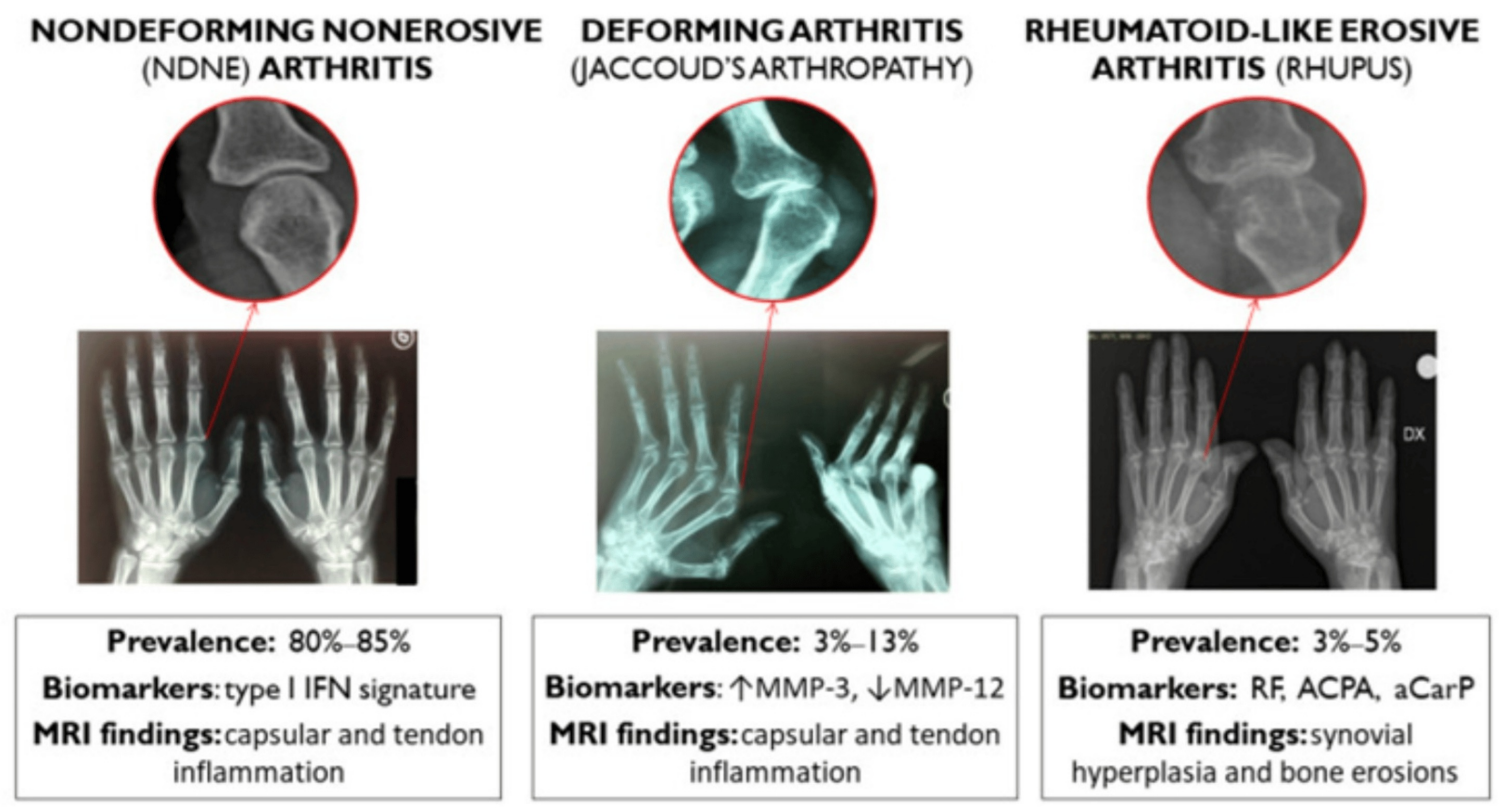
Types of systemic lupus erythematosus (SLE)-associated arthritis IFN: interferon; MMP: matrix metalloproteinase; RF: rheumatoid factor; ACPA: anti-citrullinated protein antibodies; aCarP: anti-carbamylated proteins antibodies

Musculoskeletal involvement is one of the most common manifestations of SLE, affecting up to 90% of patients. It is the initial symptom in 60-80% of cases and occurs in up to 60% of disease flares [3]. Joint issues, such as arthralgia or arthritis, are usually transient but can resemble RA, with persistent pain, swelling, stiffness, and disability. The most frequently affected joints include the metacarpophalangeal and interphalangeal joints, wrists, and knees, although tenosynovitis or tendonitis may also occur. Joint involvement in SLE can present in various clinical phenotypes: (a) joint pain (arthralgia); (b) non-deforming non-erosive (NDNE) or mildly deforming polyarthritis; (c) more rarely, radiologically non-erosive but deforming arthropathy, known as Jaccoud’s arthropathy, which occurs in 3-13% of patients; and (d) rheumatoid-like erosive arthritis (rhupus), seen in 3-5% of patients [3]. Figure 1 demonstrates the types of SLE-associated arthritis [3]. Severe JA significantly impairs joint function and increases the risk of misdiagnosis as rheumatoid arthritis [4]. Serum levels of matrix metalloproteinase (MMP)-3 are elevated, while MMP-12 levels are reduced in patients with SLE. These levels are influenced by inflammation and glucocorticoids and are associated with JA [5].

## Conclusions

This case report highlights the occurrence of JA in a young male with SLE. The patient's presentation with joint pain and characteristic deformities, along with laboratory and imaging findings, led to the diagnosis of SLE-associated JA. Early recognition and appropriate management, including the use of prednisone, diclofenac, and hydroxychloroquine, resulted in significant improvement in the patient's symptoms and a decrease in anti-dsDNA antibody levels.

JA, though less common, is a notable manifestation of SLE and can significantly impact patients' quality of life. It is important for clinicians to be aware of this condition, as its presentation can mimic other arthropathies such as rheumatoid arthritis. Proper diagnosis and treatment can lead to better patient outcomes and reduce the risk of long-term joint damage and disability. This case also highlights the importance of more research in developing diagnostic criteria that differentiate rheumatoid arthritis from Jaccoud's arthropathy, and it underscores the importance of creating disease-specific treatment plans. This case underscores the importance of comprehensive evaluation and tailored therapy in managing complex autoimmune conditions like SLE.
